# Interleukin Profiling in Atopic Dermatitis and Chronic Nodular Prurigo

**DOI:** 10.3390/ijms25158445

**Published:** 2024-08-02

**Authors:** Henning Wiegmann, Lina Renkhold, Claudia Zeidler, Konstantin Agelopoulos, Sonja Ständer

**Affiliations:** Section Pruritus Medicine and Center for Chronic Pruritus, Department of Dermatology, University of Muenster, 48149 Muenster, Germany

**Keywords:** chronic pruritus, atopic dermatitis, nodular prurigo, interleukin-4, interleukin-13

## Abstract

The clinical manifestations of atopic dermatitis (AD) and chronic nodular prurigo (CNPG) include pruritus and eczema/lesions, posing significant challenges for patients. Th2 cells and ILC2, marked by cytokine production—particularly IL-4/13—are crucial therapeutic targets. Despite displaying a dose-dependent lack of pruritus induction post-injection, IL-13 acts through the IL-13Rα1 and IL-13Rα2 receptor system. Our study focused on investigating ex vivo skin biopsies in AD (n = 17), CNPG (n = 14) and healthy controls (HC; n = 10), examining the gene expression landscape of interleukins linked with pruritus (IL-13, IL-4, IL-31) and their corresponding receptors. Compared to HC, results revealed a significant upregulation of *IL-4*, *IL-13*, and *IL-13RA1* in AD, whereas CNPG did not show increased *IL13* expression. Notably, the decoy receptor *IL-13RA2* displayed intriguing patterns, with AD showing a marked increase compared to both HC and CNPG. Positive correlations between receptor expression and itch intensity and hyperkinesis sensation underscore clinical relevance, potentially serving as biomarkers. The findings suggest a pivotal role of IL-4 and IL-13, along with IL-13RA1, in pruritus pathogenesis in both entities, while *IL-13* upregulation in AD is countered by IL-13RA2. The comparable expression of *IL-13RA2* to HC in CNPG suggests the absence of this regulatory mechanism, potentially worsening the disease and leading to prolonged scratching behavior. These insights illuminate the intricate interplay of interleukins and receptors in different pruritus phenotypes, laying the groundwork for understanding underlying mechanisms and offering avenues for therapeutic intervention.

## 1. Introduction

In the complex realm of dermatological pathology, interleukins (ILs) stand as key regulators, orchestrating the immune response within the skin’s microenvironment. In particular, in conditions like atopic dermatitis (AD) and chronic nodular prurigo (CNPG), the role of these cytokines emerges as pivotal [1]. Elevated levels of pro-inflammatory cytokines, notably IL-4, IL-13, and IL-31, pervade the epidermal landscape of individuals facing the burdens of AD and CNPG [2,3]. Such cytokines serve as architects of the immune cascade, fostering the hallmark Th2-skewed immune response characteristic of both entities and precipitating chronic inflammation while undermining the integrity of the skin barrier [4]. Emerging evidence suggests that Th2-related cytokines potentiate TRP channels, exacerbating inflammation and itch sensation, highlighting the importance these mediators [5]. Beyond their conventional pro-inflammatory roles, IL-4, IL-13, and IL-31 demonstrate a direct association with the disturbing symptomatology of pruritus, representing a distressing hallmark of AD and CNPG pathology [6]. Their nuanced involvement in the neuro-immunological interplay underlying pruritic sensations reflects the intricate pathophysiology of these dermatological conditions, underscoring the multifaceted nature of cytokine-driven inflammatory responses within the cutaneous milieu. The pronounced upregulation of IL-4 and its cognate receptor, which is a hallmark feature of the pro-inflammatory Th2 response [7], is extensively recognized in AD pathogenesis [8]. Targeting IL-4 receptor (IL-4R) signaling pathways emerges as a promising therapeutic avenue that offers hope for individuals dealing with the persistent pruritus and cutaneous inflammation characteristics of AD [9]. Intricately intertwined with that of IL-4, the IL-13 signaling cascade relies on the concerted actions of IL-4R and IL-13 receptor alpha 1 (IL-13RA1). Conversely, due to its lack of known cytoplasmic signaling motifs, IL-13 receptor alpha 2 (IL-13RA2) assumes the role of an IL-13 decoy receptor, modulating the biological effects of IL-13 within the cutaneous microenvironment [10]. Notably, the sensitizing effects of both IL-4 and IL-13 on sensory nerve fibers within human skin present a compelling story, elucidating their pivotal roles in triggering neuroinflammation and exacerbating the pruritic cascade [11]. The relevance of chronic stress and the associated infiltration of mast cells as a major source of IL-4 and IL-13 and promotor of neuronal cell death is also discussed in the context of neuroinflammation [12,13]. In order to unravel the intricacies of cytokine-mediated pathogenesis in AD and CNPG, we conducted a small exploratory study using patients diagnosed with AD (n = 17), CNPG (n = 14), and healthy control subjects (HC, n = 10). The study sought to elucidate the gene expression profiles of IL-4, IL-13, IL-31, and their corresponding receptors, namely, IL-4R, IL-13RA1, IL-13RA2, and IL-31RA, in whole-skin samples obtained from the study cohort.

## 2. Results

**Figure 1:**
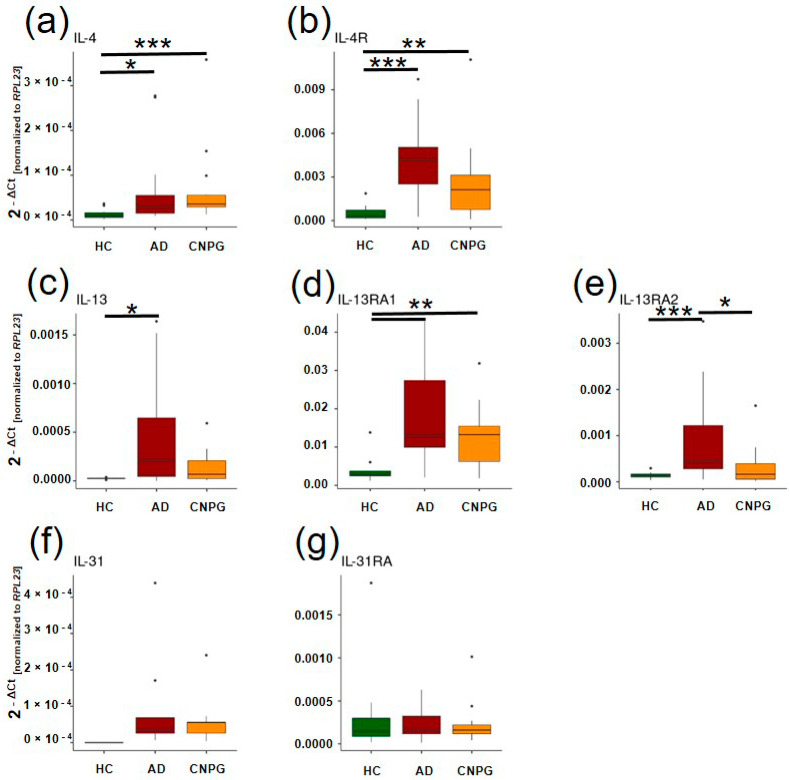
Gene expression analyses in skin of patients with AD and CNPG (**a**–**g**). Gene expression of the interleukins *IL-4*, *IL-13*, *IL-31*, as well as their receptors *IL-4R*, *IL-13RA1*, *IL-13RA2*, *IL31RA*. For statistics, non-parametric Mann–Whitney U test was used. The significance levels were set as * *p* < 0.05, ** *p* < 0.005, *** *p* < 0.001. IL: interleukins, AD: atopic dermatitis, CNPG: chronic nodular prurigo, HC: healthy controls, dots represent outliers.

The investigation into the molecular landscape of atopic dermatitis (AD) and chronic nodular prurigo (CNPG) unveils a complex interplay of interleukins (ILs) and their receptors, shedding light on the intricate mechanisms underpinning these dermatological conditions. Elevated expression levels of IL-4 and its receptor (IL4R) were notably observed in both AD and CNPG cohorts—*IL-4* (AD median 2.88 × 10^−5^ IQR [1.49 × 10^−5^; 5.50 × 10^−5^] > HC, *p* < 0.05; CNPG 3.58 × 10^−5^ [2.90 × 10^−5^; 5.51 × 10^−5^] > HC, *p* < 0.001) and *IL4R* (AD 4.17 × 10^−3^ [2.50 × 10^−3^; 5.04 × 10^−3^] > HC, *p* < 0.001; CNPG 2.12 × 10^−3^ [7.64 × 10^−4^; 3.12 × 10^−3^] > HC, *p* < 0.005; Figure 1a,b)—compared to the healthy controls (HC *IL-4*: 9.33 × 10^−6^ [5.75 × 10^−6^; 1.60 × 10^−5^], *IL-4R*: 3.03 × 10^−4^ [1.75 × 10^−4^; 7.11 × 10^−4^]). There were no significant differences between the entities in *IL-4* and *IL-4R* expression (Figure 1a,b). Furthermore, patients with AD showed a significantly higher expression of *IL-13* (2.06 × 10^−4^ [4.53 × 10^−5^; 6.47 × 10^−4^], *p* < 0.05), as well as *IL-13RA1* (1.28 × 10^−2^ [9.92 × 10^−3^; 2.73 × 10^−2^], *p* < 0.005) and *IL-13RA2* (4.45 × 10^−4^ [2.81 × 10^−4^; 1.21 × 10^−3^], *p* < 0.001), compared to HC (*IL-13*: 2.44 × 10^−5^ [2.36 × 10^−5^; 2.58 × 10^−5^], *IL-13RA1*: 2.79 × 10^−3^ [2.43 × 10^−3^; 3.74 × 10^−3^], *IL-13RA2*: 1.35 × 10^−4^ [1.05 × 10^−4^; 1.70 × 10^−4^]). CNPG merely showed significantly increased *IL-13-RA1* expression (1.32 × 10^−2^ [6.24 × 10^−3^; 1.54 × 10^−2^], *p* < 0.005) compared to HC. Interestingly, the significant downregulation of *IL-13RA2* in CNPG was observed compared to AD (*p* < 0.05) (Figure 1c–e).

**Figure 2:**
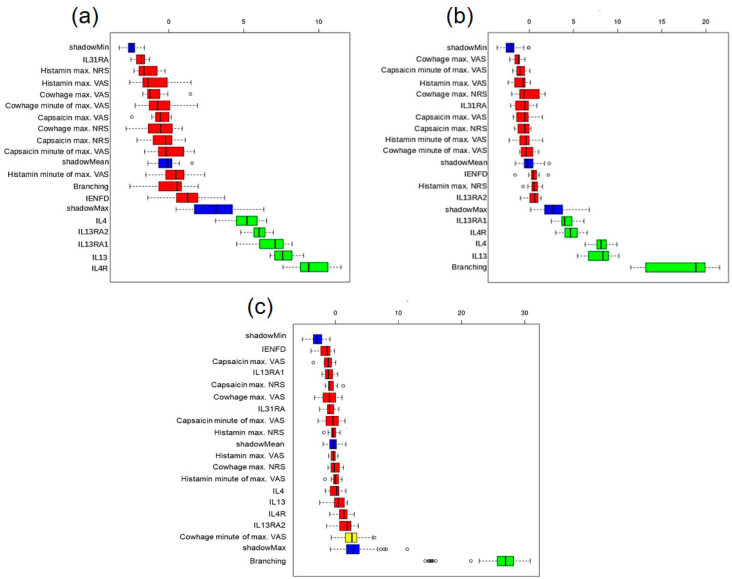
Feature importance analysis of the collected parameters using Boruta algorithm for comparison of (**a**) AD vs. HC, (**b**) CNPG vs. HC, (**c**) AD vs. CPNG (in green: important parameters, blue: algorithm-related shadow values, red: unimportant parameters, in yellow: tentative features). IL: interleukins, AD: atopic dermatitis, CNPG: chronic nodular prurigo, HC: healthy controls, IENFD: intraepidermal nerve fiber density, NRS: Numerical Rating Scale, VAS: visual analog scale, circles indicate outliers.

To investigate a potential functional role of gene expression from the present study in pruritus pathogenesis by interacting with the peripheral nerve system, we performed a meta-analysis correlating gene expression with previously published epidermal neuroanatomical data [14,15] and pruritus perception ratings after chemically induced pruritus using cowhage, histamine, and capsaicin [14]. Here, AD showed a significant positive correlation between the visual analog scale (VAS) of the last 4 weeks and *IL-13RA1* expression (r = 0.53, *p* < 0.05). There were also significant positive correlations between the VAS of the last 4 weeks and the strongest perception after chemical pruritus induction by all three treatments. In CNPG, VAS (r = 0.21, *p* < 0.05) showed a significant but small positive correlation with *IL-31RA* expression. Regarding *IL-31*, we detected expression in both AD and CNPG, whereas no expression was detectable in the healthy controls (Figure 1f). Due to the undetectable expression in HC, no statistical analysis of *IL-31* expression was performed. The expression of *IL-31RA* was found to be slightly increased in AD and reduced in CNPG compared to HC without any significance (Figure 1g). There was also a positive correlation between the expression of *IL-4* and *IL-13*, but it was not significant. Furthermore, no significant correlations were found for the sensations after chemically induced pruritus. Based on these results, we performed a decision-tree-based feature importance analysis of the parameters using the Boruta algorithm to identify parameters that are most essential for discriminating between AD/HC, as well as CNPG/HC and between AD/CNPG. Here, the expression of *IL-4*, *IL-13*, *IL-4R*, *IL-13RA1*, and *IL-13RA2* were the most important parameters in AD compared to HC. Neither the neuroanatomy nor the hyperkinesis assessments seem to be decisive and rather represent the consequence of differentially regulated marker genes (Figure 2a). Instead, the branching behavior of IENF, as the most important parameter, becomes the focus of attention again (Figure 2b). Branching was only identified as an important parameter for differentiating between AD and CNPG (Figure 2c).

## 3. Discussion

In our comprehensive study, the intriguing observation of the absence of upregulation in *IL-13RA2* expression among chronic nodular prurigo (CNPG) patients prompts an insightful exploration into the regulatory mechanisms governing cytokine signaling in CNPG. In human skin, both IL-4 and IL-13 have sensitizing effects on sensory nerve fibers and, thus, can trigger neuroinflammation and pruritus [11]. Furthermore, in mice, an injection with IL-4, IL-13, and a combination of both led directly to acute pruritus [14,16]. In response to associated pruritus, there is prominent scratching behavior that leads to basophil-dependent IL-4 upregulation [17], but may lead to increased expression of the IL-13-decoy receptor IL-13RA2 and interruption of the IL-4/IL-13 signaling cascade as well [10,18]. In our study, the lack of upregulation of *IL-13RA2* expression in CNPG patients, which was even significantly lower than in AD (*p* < 0.05), suggests the absence of this regulatory mechanism in CNPG. However, the multifaceted role of IL-13RA2 remains subject to contentious debate. Notably, in sensory neurons, IL-13RA2 can act through Toll-like receptor 2 (TLR2), as demonstrated in vitro, potentially culminating in increased IL-13-induced scratching behaviors in murine models [19]. Increased attention is necessary to unravel these complexities, especially concerning various pruritic diseases and the sensitization of peripheral nerves.

Characterized by heightened sensitivity to moderate pruritic stimuli, hyperkinesis signifies a pivotal aspect of pruritus perception, wherein a typically mild itch sensation is perceived as significantly more intense [20,21]. The intricate mechanisms underlying hyperkinesis encompass both peripheral and central nervous system components [22,23]. Notably, existing evidence suggests that IL-4 and IL-13 directly contribute to peripheral sensitization by modulating the responsiveness of peripheral nerve fibers [24,25,26].

Upon analyzing the pivotal parameters extracted in CNPG compared to healthy controls, the expressions of *IL-4*, *IL-13*, *IL-4R*, and *IL-13RA1* emerge as decisive factors, while the IL-13 decoy receptor *IL-13RA2* played no role.

In summary, our study unveils distinct gene expression patterns in AD and CNPG, underscoring the dysregulation of IL-4 and IL-13 signaling cascades and reaffirming the significant involvement of interleukins IL-4 and IL-13 in the pathogenesis of both conditions. The conspicuous absence of a regulatory mechanism involving IL-13RA2 in CNPG suggests a disruption of homeostasis, perpetuating chronic pruritus and prolonged scratching behavior. Further, correlation analyses elucidate the intricate connections between IL-4, IL-13 receptors, and pruritus perception, shedding light on their potential role in peripheral sensitization. Moreover, the application of the Boruta algorithm identifies *IL-4*, *IL-13*, *IL-4R*, *IL-13RA1*, and *IL-13RA2* as indispensable parameters distinguishing AD and CNPG from healthy controls. These findings underscore the complex immunological mechanisms, underpinning these dermatological conditions and offering invaluable insights into potential avenues for targeted therapeutic interventions aimed at mitigating the burden of pruritic symptoms. However, the small cohort size of our study represents a limitation and must be taken into account when interpreting the results.

## 4. Materials and Methods

### 4.1. Patient Recruitment and Testing of Chemically Induced Hyperknesis

This analysis was conducted on the basis of a broader study in which patients were recruited and the clinical parameters and sensation tests after chemically induced hyperkinesis were recorded. The patients included were chronic pruritus patients (pruritus duration > 6 weeks) in an acute exacerbation episode [13,14]. Due to the limitation of RNA availability, further gene expression analyses were carried out on a small sub-cohort of the broader study mentioned above. Only data from this sub-cohort were used for subsequent analyses (Table 1). The study was approved by the local ethics committee of Westfalen-Lippe (2011-114-f-S).

### 4.2. Gene Expression Analysis

For the analysis of gene expression, RNA was isolated (AllPrep DNA/RNA/Protein Kit, Qiagen, Hilden, Germany) from whole-skin biopsies and 400 ng RNA of each sample was transcribed into cDNA. TaqMan assays from Thermofisher Scientific (Karlsruhe, Germany) were used (Table 2), as well as the qPCR cycler Quantstudio 1 (Thermofisher Scientific, Karlsruhe, Germany). Normalization was performed using the housekeeping gene *RPL23*. The 2^−Ct^ values were used for the graphical representation.

### 4.3. Data Analysis and Statistics

The non-parametric Mann–Whitney U test was used for the statistical analysis of the independent samples. Values of gene expression are the median, and the interquartile range (IRQ) is given in square brackets. The Spearman rho coefficient was calculated for the correlation analyses. The significance levels were set as * *p* < 0.05, ** *p* < 0.005, and *** *p* < 0.001. Both the calculations and the visualizations were performed with the software R version 4.3.2 (31 October 2023). Missing values were replaced by the median in the case of numerically scaled parameters, while missing ordinal scaled parameters were replaced by the mode value. The percentage of missing values in the dataset was less than 5%. For the analysis of important features for classification, the Boruta algorithm was used. The Boruta algorithm is a feature selection method primarily employed in machine learning classification tasks [27].

The algorithm introduces “shadow features” by permuting values within each feature column of the dataset, creating a reference for comparison. Feature importance is determined by training a Random Forest on the original dataset and assessing each feature’s contribution to model accuracy.

Comparison with shadow features helps identify relevant features, and a significance test determines the statistical significance of their importance. The process iterates, refining feature selection until a stopping criterion is met. Boruta categorizes features as “important”, “unimportant”, or “tentative”. The final list of selected features is then provided, with tentative features indicating uncertainty. The following R packages were used for the analysis: rpart.plot_3.1.1, rpart_4.1.23, patchwork_1.2.0, factoextra_1.0.7, Boruta_8.0.0, PerformanceAnalytics_2.0.4, ggpubr_0.6.0, ggcorrplot_0.1.4.1, magrittr_2.0.3, tidyverse_2.0.0.

## Figures and Tables

**Table 1:** Demographic data and pruritus characteristics of the patient cohort.

Diagnosis	Sex (m/f)	Mean Age [min/max]	Median VAS Last 4 Weeks [IQR]	Median Pruritus Duration in Month [IQR]
Atopic dermatitis	8/9	50.06 [21; 71]	7 [5; 8]	133.35 [70.98; 281.19]
Chronic nodular prurigo	3/11	56.5 [37; 76]	5 [2.5; 7]	101.11 [63.71; 183.88]
Healthy controls	6/4	49.3 [23; 66]	NA	NA

VAS: visual analog scale; m: male; f: female; IQR: interquartile range, NA: not applicable.

**Table 2:** TaqMan assays used for gene expression analysis.

Gene	Name	Amplicon Length (bp)	Company
*IL4*	Hs00174122_m1 interleukin 4 TaqMan Assay	70	Thermo Fisher Scientific
*IL13*	Hs00174379_m1 interleukin 13 TaqMan Assay	82	Thermo Fisher Scientific
*IL4R*	Hs00166237_m1 interleukin 4 receptor TaqMan Assay	70	Thermo Fisher Scientific
*IL13RA1*	Hs00609817_m1 interleukin 13 receptor subunit alpha 1 TaqMan Assay	147	Thermo Fisher Scientific
*IL13RA2*	Hs00152924_m1 interleukin 13 receptor subunit alpha 2 TaqMan Assay	83	Thermo Fisher Scientific

IL: interleukin; R: receptor; RA: receptor subunit alpha; bp: base pairs; Hs: *Homo sapiens*.

## Data Availability

Data is contained within the article.
